# Grief Reactions and Grief Counseling among Bereaved Chinese Individuals during COVID-19 Pandemic: Study Protocol for a Randomized Controlled Trial Combined with a Longitudinal Study

**DOI:** 10.3390/ijerph18179061

**Published:** 2021-08-27

**Authors:** Renzhihui Tang, Tong Xie, Keyuan Jiao, Xin Xu, Xinyan Zou, Wenli Qian, Jianping Wang

**Affiliations:** 1 Faculty of Psychology, Beijing Normal University, Beijing 100875, China; 201831061020@mail.bnu.edu.cn (R.T.); 202031061021@mail.bnu.edu.cn (T.X.); 201731060009@mail.bnu.edu.cn (X.X.); zouxinyan@mail.bnu.edu.cn (X.Z.); 202021061102@mail.bnu.edu.cn (W.Q.); 2 Department of Social Work and Social Administration, The University of Hong Kong, Hong Kong 999077, China; kyjiao@connect.hku.hk

**Keywords:** grief reactions, mental health, bereavement, grief counseling, COVID-19 pandemic, prolonged grief disorder, complicated grief

## Abstract

COVID-19 has caused nearly 4.3 million deaths all around the world. People who have experienced loss during this special period may find it difficult to adapt to life after loss, and may even suffer from prolonged grief disorder or other mental health problems. However, there is a huge gap of grief research in China, with almost no comprehensive grief intervention training system or very few professional grief consultants. Considering the large number of bereaved individuals who are suffering from grief and other mental health problems, it is significant to develop a suitable and effective intervention protocol immediately. This article illustrates a study protocol initiated by a Chinese university to investigate the mental health of bereaved individuals during the COVID-19 pandemic and train grief counselors to provide grief counseling to the bereaved, as well as to evaluate the effectiveness of the grief counseling. The method is as follows: (1) 300 psychological counselors will be recruited to attend the grief counseling training. Assessments will be conducted at three time points: baseline (T0), after the basic training (T1), and after the advanced training (T2); (2) 500 bereaved Chinese will be recruit to join the online survey and will be assessed at two time points with a six-month interval; and (3) a two-armed (grief counseling versus wait-list controls) RCT (random control trials) will be conducted with 160 bereaved individuals. Assessments will be conducted at three time points: before randomization (baseline, T0), at the post-counseling (T1), and three months after the post-counseling (T2). Primary outcomes will be assessed by the Prolonged Grief Questionnaire (PG-13), the 20-item PTSD Checklist for DSM-5 (PCL-5), the Depression Anxiety and Stress Scale (DASS-21), and the Posttraumatic Growth Inventory (PTGI). This research will help develop grief research and grief counseling in China, as well as provide professional mental health services for individuals who may suffer from grief-related disorders in the future.

## 1. Introduction

In December 2019, Coronavirus Disease 2019 (COVID-19) was first identified in Wuhan, China. As of 18 August 2021, COVID-19 has caused more than 4.3 million deaths all around the world [1], creating an unprecedented pandemic. A recent study showed that about nine family members would be affected by each death from COVID-19 [2]. Based on this estimate, there will be a huge number of the newly bereaved after COVID-19. In addition, bereaved individuals who have lost their loved ones during the pandemic are also faced with more difficult situations than usual (social isolation, lack of traditional memorial ritual, shortage of medical resources, etc.). Therefore, it is exceptionally urgent to provide appropriate and professional grief counseling or grief therapy for bereaved individuals during this period.

### 1.1. Bereavement and Grief Reactions

Grief is the natural response to losing a loved one. Most individuals adapt to their loss in natural ways. However, there exist substantial variations between individuals in terms of adjustments to loss [3]. In fact, various responses to loss tend to follow several distinct trajectories [4]. After the initial period of losing a loved one, some people could gradually adapt to the new life and move on with their love and memories of the deceased. For others, however, their process of adjusting to the grief is interfered. For a few people, negative mental and physical health consequences are extreme and persistent after bereavement. Individual factors such as maladaptive thoughts, dysfunctional behaviors, or inadequate emotion regulation can largely impede this process [5]. The sudden and unexpected death of a significant individual in one’s life is a highly intense stress event. It can trigger a series of grief reactions, including changes in emotion, cognition, behavior, and physiology [6].

With regard to emotional reactions, the bereaved may have feelings of depression, anxiety, anger, despair, loneliness, longing, and guilt; in terms of cognitive reactions, there may be rumination, self-blame, low self-evaluation, and meaninglessness. At the same time, the bereaved may show behavior reactions such as crying, nervousness, social withdrawal, and repeated search for the deceased. In addition, there may also arise some physiological problems, such as loss of appetite, insomnia, and fatigue [4,6,7,8,9,10]

For most of the bereaved, they can gradually accept the reality of loss and adapt to new life without the deceased under the influence of some factors, such as adequate social support, traditional funeral rituals, and meaning reconstruction [11]. However, studies have suggested that some bereaved people may develop intense and continuous symptoms in emotional, cognitive, behavioral, and physiological aspects, which may lead to the impairment of their mental or physical health and social functions [6,7,10,12].

The topic of grief and loss is a common issue for people all around the world [13]. The establishment of diagnostic criteria will facilitate the development of research and practice in grief field, and the diagnostic criteria applicable to clinical and practical applications will be beneficial to improve clinical utility [14].

In 2018, the 11th edition of the World Health Organization’s new International Classification of Diseases (ICD-11) established prolonged grief disorder (PGD) as one of the stress-related diseases [15]. According to the ICD-11, the diagnosis of prolonged grief disorder should conform to the following criteria: six months after the bereavement, longing for the deceased and/or persistent preoccupation with the deceased, accompanied by strong emotional pain, such as grief, guilt, anger, denying, blaming oneself or others, having difficulty accepting that relatives and friends have passed away, feeling that part of one’s self has passed away with the departure of relatives and friends, unable to experience positive emotions, and emotional numbness [15]. And the symptoms should reach a level where the grief seriously affects one’s own life, work, and social interactions. An important goal of diagnostic evaluation is to identify patients who need support and to provide recommendations on the available treatment [14]. The new diagnosis of PGD in ICD-11 shows a flexible guideline to allow space for more clinical judgement and a classification system with fewer categories [14,16]. It has played a key role in promoting attention to the mental health of bereaved individuals and the development of targeted treatment programs for patients with prolonged grief disorder after bereavement. [16,17].

In addition to prolonged grief disorder, which is a bereavement-specific psychological risk, previous studies have also found that bereaved individuals show increased risk of suffering other mental disturbances and health problems [18], such as depressive disorder [19,20], anxiety disorder [21], post-traumatic stress disorder [22,23], self-harm problems [24], suicidal behavior [25], sleep disorders, substance abuse, immune dysfunction [5], cardiovascular disease and cancer risk. Among bereaved individuals, the prevalence of post-traumatic stress disorder is as high as 10–30%, and there exists a complicated comorbidity with prolonged grief disorder [26,27].

### 1.2. Bereavement and Grief under COVID-19 Outbreak

According to previous studies, the prevalence of prolonged grief disorder in the general bereaved population is approximately 9.8% [28]. Sudden and violent deaths have the potential to lead to a higher prevalence of PGD among the bereaved, from anywhere between 14% and 76% [29].

In this pandemic, bereaved individuals are faced with countless difficulties and challenges. Due to the contagious nature of the virus, relatives are often unable to visit, take care of, or even check in on the patients. Many people experience this loss without preparation. In addition, due to the quarantine policy, it is impossible to hold the traditional Chinese family-gathering memorial ritual after the death. The life of the majority of bereaved individuals have been greatly affected by the virus, and some bereaved individuals have even experienced the loss of multiple relatives at the same time. The aforementioned difficulties, such as unexpectedness, no farewell rituals, multiple losses, and lack of self-security and so on, may lead to a higher risk of mental disturbances (such as prolonged grief disorder) than usual. A recent study indicated that during COVID-19, the prevalence of anxiety or depression was 20.4% [30]. The high levels of mental health problems may hinder the process of grief adaptation. According to a recent study, researchers found that over one-third of COVID-19 related bereaved Chinese individuals suffered from PGD [31]. The high prevalence demonstrated in the study above validates the importance and urgency of the concerns and early interventions for bereaved individuals due to COVID-19.

### 1.3. Grief Counseling and Grief Therapy

We often say “time will cure everything”. However, for individuals with prolonged grief disorder, they may experience a stronger grief response over time, without the help of professional grief counselors [32]. Moreover, the prolonged grief disorder does not only lead to emotional pain and impaired social function for the patients themselves, but also brings heavy stress and pain to other relatives.

Grief is a natural experience after loss, but for those bereaved who have difficulty adapting to grief, seeking help from psychotherapy is important [33]. Consequently, more research is needed to explore short-term, evidence-based, grief-focused, and effective psychological treatments.

The present grief treatments include: (a) meaning reconstruction [34], emphasizing that reconstruction of lost meaning is an important process of grief adapting; (b) complicated grief treatment (CGT) [5], focusing on the two directions of coping with loss and rebuilding a new life; (c) cognitive-behavioral therapy for complicated grief (CBT-CG) [35], helping individuals to integrate loss experiences into their autobiography through imaginal exposure, cognitive restructuring, and behavioral activation, while reducing negative self-evaluation, dysfunctional beliefs, and their avoidance behavior, and finally achieving the purpose of helping them better adapt to their life after loss.

### 1.4. Grief Research and Grief Counseling in China

There is hardly any systematic grief intervention training system and professional grief consultants in China. In emergencies of major public health crises, the Chinese government often ignores the psychological aspect of the rescue of the people, especially the psychology support for the bereaved. Faced with characteristics of the grief among the bereaved from this pandemic, the gap in grief intervention is particularly prominent. During COVID-19, there may be a great many bereaved individuals who have difficulty adapting to their loss. However, due to the insufficient number of professional psychological counselors, social isolation, and other reasons, the majority of bereaved Chinese can’t gain access to reliable psychology resources. Although western countries have a relatively complete bereavement support and grief counseling system, as well as a professional training system for grief counselors, it remains to be established through empirical studies if it is the case that western protocols are feasible to apply to the situation in China.

### 1.5. Rationale for the Research

In view of the urgency of mental health assistance for the bereaved during COVID-19, we set up a team and designed this present research. Notably, this research is not only a scientific research project, but also a social welfare project. Whether or not the bereaved research participants are willing to have their data collected for this research, we will provide free grief consulting services for more than 160 bereaved individuals during the research period.

The first aim of this project is to train and evaluate Chinese grief consultants with reference to the more developed western grief consultant training system, and to fill the huge gap of grief consulting in China. Secondly, previous studies suggest that related factors (such as unexpected death, social isolation, and multiple losses) may hinder the process of adaptation and recovery of bereaved individuals, but the mechanism remains to be elucidated, so it is necessary to design a longitudinal study to investigate the mental health and associated factors of the bereaved in China [31]. Finally, we set out to develop a grief counseling program suitable for Chinese culture to provide support for bereaved people during COVID-19, while verifying the effectiveness of the design, and supplementing grief research.

The principal research objectives are to:

1. Investigate demographic and related factors associated with prolonged grief symptoms among Chinese individuals bereaved due to COVID-19 (including grief reaction, trauma response, depression, anxiety, and suicide risk).
2. Develop training and evaluation programs for Chinese professional grief counselors to develop and examine their competence.
3. Provide grief counseling for the bereaved during the pandemic and assess the effect of the intervention.

## 2. Research Design

**Figure 1:**
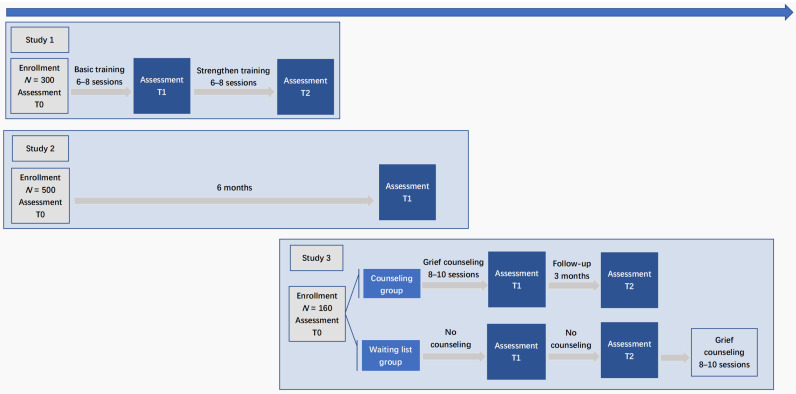
Study design.

This research includes three studies, and the overall research framework is shown in Figure 1.

### 2.1. Study 1: Grief Counselor Training and Evaluation

Study one aims to professionally train psychological counselors to be competent to carry out grief counseling. 300 candidates for the program will be recruited. Certain information will be gathered from the candidates, who will be screened for suitability for the program. Training program targeting grief counseling will be designed and implemented to coach these counselors. The training program consists of a basic phase and an advanced phase. A certain competency test will be carried out before the training, and after the two phases of the training to evaluate the effectiveness of the training program.

#### 2.1.1. Sample

Participants can be included in this study if they meet the following criteria:

Educational background: Master’s degree in psychology (consulting and clinical psychology), psychiatry, nursing, or social work.

• Qualifications: (a) Qualified as a registered psychologist, registered social worker, or occupational psychological counselor (national second level); (b) provided more than 500 h of psychological consultation for clients and received at least 50 h of individual supervision or 100 h of group supervision; and (c) received psychological counseling ethics training in the past three years.
• Voluntary participation in the whole process of research.
• In addition, the consultants who sign up are required to provide the following information for screening: (a) experiential description of grief counseling services; (b) certificate of participating in systematic psychological counseling or treatment training; (c) experience in grief counselling training, crisis intervention training, trauma-related training; (d) personal grief experience; (e) willingness and motivation to participate in grief counseling.

#### 2.1.2. Procedure

A competency test will be given to all counselors who have passed the screening before the training (T0). The competency test will consider three dimensions of competency (knowledge, attitude, and practice) and draw on the competency instrument of the training program of the Complicated Grief Research Center of Columbia University, and it will be adjusted to the context of Chinese psychotherapy. A training program will be designed with the experience from domestic and foreign experts in the field of grief, then a six to eight sessions training will be given to all counselors in basic phase, and advanced phase respectively. After the basic (T1) and advanced (T2) training, the counselors will be assessed again with the same competency test.

### 2.2. Study 2: Prevalence and Related Factors of Prolonged Grief Disorder of Bereavement in COVID-19

This study intends to longitudinally explore the characteristics of mental health and demographic and loss-related factors associated with prolonged grief symptoms among bereaved Chinese individuals during COVID-19 pandemic. The researchers are concerned with not only the level of grief reactions (between-individual differences) but also its change (within-individual differences). About 500 bereaved Chinese will be recruited online.

#### 2.2.1. Sample

Participants can be included in this study if they are over the age of 18 and have lost their first-degree relatives during COVID-19.

Exclusion criteria consists of a lifetime history of severe mental disorder (e.g., schizophrenia, bipolar disorder); intellectual disability; alcohol or substance abuse or dependence within the past six months; strong suicidal intention.

#### 2.2.2. Procedure

An online link will be sent to the participants who meet the inclusion criteria. Once the participants access the link, the information concerning the research purpose, and the informed consent form, will appear. Only when the participants select “I understand the above information and agree to participate in this research,” will they begin the formal study. At the end of the survey, the researcher will provide the participants with a supporting resource package (including hotline number, self-help resources, and related websites). Assessments will be conducted at two time points: baseline (T0) and six months after the baseline (T1).

#### 2.2.3. Measures

In order to alleviate the discomfort that the bereaved may feel when filling in the questionnaires, the researchers will try to reduce the number of the items as much as possible. Table 1 shows all the measures that will be carried out at each time point.

##### Demographic and Loss-Related Information

Demographic information includes age, sex, education level, marital status and religious belief. Loss-related information includes the relationship with the deceased, time since the loss, and the cause of death.

##### Primary Outcomes

Prolonged Grief Questionnaire (PG-13) [8] and Inventory of Traumatic Grief (ITG) [36] will be used to measure the grief symptoms of the bereaved. Post-traumatic stress symptoms will be assessed by the 20-item PTSD Checklist for DSM-5 (PCL-5) [37]. Depression Anxiety and Stress Scale (DASS-21) [38] will be used to measure the depressive symptoms, anxiety symptoms, and stress status of the bereaved. Posttraumatic Growth Inventory (PTGI) [39] will be used to assess the perceived benefits from traumatic events.

##### Secondary Outcomes

The Scale for Suicidal Intention (SSI) [40] will be used to measure the severity of suicidal intentions. Maladaptive cognitions common in the bereaved will be measured by the Typical Beliefs Questionnaire (TBQ) [41]. The Grief-related Avoidance Questionnaire (GRAQ) [9] will be used to assess the avoidance behaviors of the bereaved. The Work and Social Adjustment Scale (WSAS) [42] will be used to assess the participants’ functioning in work, social adjustment, management, and family relationships.

### 2.3. Study 3: Grief Counseling for Bereaved Chinese during COVID-19

Based on the above grief therapy description, combined with the results of study two, the researchers will develop an online grief counseling plan that is more suitable for Chinese culture. The counseling is expected to include 8–10 sessions (one hour per session). The themes of each of the sessions are: (1) understanding and accepting grief reactions; (2) managing painful emotions; (3) learning to care for yourself; (4) increasing contact with others; (5) coping with difficult days; and (6) adapting to a new life.

#### 2.3.1. Sample

One-hundred-sixty bereaved Chinese will be recruited online. The inclusion criteria in study three are the same as study two.

#### 2.3.2. Procedure and Measures

The researchers aim to conduct a single-blinded randomized controlled trial (RCT) evaluating the effectiveness of grief counseling versus a waitlist control group in reducing prolonged grief symptoms, post-traumatic symptoms, depression levels, and suicidal intentions in bereaved Chinese individuals in relation to COVID-19.

After finishing the baseline questionnaires and signing the informed consent, 160 bereaved participants will be randomly assigned to the grief counseling group or the waitlist group.

The sample size is calculated based on the expected difference on the primary outcome variable (grief symptoms of the bereaved) between the different groups. According to previous RCTs on bereaved individuals [43], we expect a medium Cohen effect size of 0.5 in the intervention group. The sample size calculation is based on the conventional significance (α) and power (1−β) levels of 0.05 and 0.80, respectively, and an expected dropout percentage of 20%. The result shows that we will need almost 80 participants for each group, therefore, we have determined the total sample size to be 160 in this study.

The measures in study three are the same as study three. Assessments will be conducted at three time points: before randomization (baseline, T0), at post-counseling (T1), and three months after the post-counseling (T2).

Table 2 shows which measures will be assessed at each time point. And Figure 1 shows the research process.

## 3. Statistics Analyses

IBM SPSS Version 25 (Armonk, New York, USA) will be used to analyze the data. Both completer analyses and intention-to-treat analyses (using the method of last observation carried forward) will be carried out. Descriptive characteristics of the sample will be calculated as obtained counts (*n*) and simple proportions (%), with missing items reported as missing.

For study one, the measurements at three different time points will be compared using the analyses of variance (ANOVA) for continuous variables and the chi-square test for categorical variables.

In addition, in study two, the researchers will employ linear regression analyses to examine the predictors of PG-13, DASS-21, PCL-5 and PTGI at each time point, while controlling for gender, age, education level, marital status, and religious beliefs. The cross-lagged model will be used to further clarify the direction of the relationship between the predictors and outcome variables. A *p*-value < 0.05 (2-tailed) will be taken to indicate statistical significance.

For study three, Chi-square tests and ANOVAs will be conducted on baseline scores to rule out significant differences between groups on demographic or loss-related or outcome variables before treatment. The treatment effects on the primary outcome variables will be examined with a 2 (groups) × 3 (time points) multivariate analysis of variance (MANOVA), with three time points (baseline, post-counseling, and three months follow-up) as the repeated measures. The outcomes of treatments will be further investigated separately with the analyses of covariance (ANCOVA), using baseline scores of the outcome variables as covariates.

## 4. Discussion

Increasing attention has been paid to bereavement-related disorders in recent decades. Grief is an inevitable experience for each human being. But adjustment to loss has substantial variations between individuals, and various responses to loss follow several distinct trajectories [4,8]. After the initial period of losing a loved one, some people may gradually adapt to their new life and move on with the love and memories of the deceased; for others, however, there are some factors that may hinder their adjusting process to grief; and for a few people, negative mental and physical health consequences may be extreme and persistent after loss [4,32]. Grief resulting from the loss of a loved one due to COVID-19 may be complicated with several factors (e.g., experiencing multiple losses, lack of traditional rituals) and may thus prove to be more persistent or extreme.

The trajectories of bereavement could be affected by socio-cultural factors. According to previous research, bereaved parents in China presented different patterns of grief symptoms (e.g., higher levels of loneliness, emptiness, and numbness) compared to Swiss bereaved parents [44,45]. Therefore, it is necessary to explore grief reactions and assess the effectiveness of grief therapy in different cultural backgrounds. This research will provide valuable insight into grief research and grief counseling in China, as well as providing professional mental health services for individuals who may develop grief-related disorders in the future.

Considering the large number of bereaved individuals who are suffering from grief and other mental health problems, it is significant to develop a suitable and effective intervention protocol immediately. With this research, we hope to help fill in the gap in the research and therapy of bereaved individuals during COVID-19 in China.

To the best of our knowledge, this will be the first time that such a large-scale grief research project will have been conducted among bereaved individuals in mainland China during COVID-19 pandemic. This project will carry positive implications for practice, research, policy.

In terms of practical significance, this project will be beneficial to increasing understanding of the mental health status of the bereaved individuals, and promoting awareness of mental health in China. Since there are very few professional grief counselors in mainland China, one of the aims of this project is to provide grief theory and grief counseling training to experienced counselors, to improve their competence, and provide professional grief counseling services for bereaved individuals in the future. This research develops, conducts, and evaluates the training program of grief counselling in mainland China for the first time, which will largely promote the development of grief counseling in China.

From a research perspective, as far as we know, this is the first study in mainland China to explore the training effect of grief counselors, and to evaluate the efficacy of RCT grief counseling for bereaved individuals. This study is rooted in China’s local culture and policy conditions. This study may improve grief theories under the background of Chinese culture, expands the depth and breadth of grief research culturally, and also provides theoretical support for further grief research and grief intervention.

As for policy perspective, China’s mental health work is still in its infancy. Our project may help raise awareness of the area of grief, and promote the development of policies for the physical and mental health of the bereaved individuals.

## 5. Ethics, Consent and Permissions

This research will follow the latest version of the Declaration of Helsinki. The study protocol was approved by the Institutional Ethics Committee of Beijing Normal University and the Chinese clinical trial registry has approved the study (Register ID: ChiCTR2000038255). Informed consent to participate in the study will be obtained from all participants. Participants will be informed that they can withdraw at any time from the study without consequences.

## Figures and Tables

**Table 1:** Schedule of assessments.

Measures	Baseline	Follow-Up (3 Months after Baseline)
Socio-demographic information	×	
PG-13	×	×
ITG	×	×
PCL-5	×	×
PTGI	×	×
DASS-21	×	×
TBQ	×	×
GRAQ	×	×
WSAS	×	×
SSI	×	×
Therapy expectations	×	×

PG-13 = Prolonged Grief Questionnaire; ITG = Inventory of Traumatic Grief; PCL-5 = PTSD Checklist for DSM-5; PTGI = Posttraumatic Growth Inventory; DASS-21 = Depression Anxiety and Stress Scale; TBQ = Typical Beliefs Questionnaire; GRAQ = Grief-related Avoidance Questionnaire; WSAS = Work and Social Adjustment Scale; SSI = the Scale for Suicidal Intention.

**Table 2:** Schedule of assessments.

Measures	Baseline (Before Randomization)	Post-Counseling	Follow-Up (3 Months after the Post-Counseling)
Socio-demographic information	×		
PG-13	×	×	×
ITG	×	×	×
PCL-5	×	×	×
PTGI	×	×	×
DASS-21	×	×	×
TBQ	×	×	×
GRAQ	×	×	×
WSAS	×	×	×
SSI	×	×	×
Therapy expectations	×		

PG-13 = Prolonged Grief Questionnaire; ITG = Inventory of Traumatic Grief; PCL-5 = PTSD Checklist for DSM-5; PTGI = Posttraumatic Growth Inventory; DASS-21 = Depression Anxiety and Stress Scale; TBQ = Typical Beliefs Questionnaire; GRAQ = Grief-related Avoidance Questionnaire; WSAS = Work and Social Adjustment Scale; SSI = the Scale for Suicidal Intention.

## Data Availability

The study did not report any data.
